# Toxicological studies of stem bark extract from *Schefflera barteri* Harms (Araliaceae)

**DOI:** 10.1186/s12906-015-0581-z

**Published:** 2015-03-07

**Authors:** Serge Secco Atsafack, Jules-Roger Kuiate, Raymond Simplice Mouokeu, Martin Luther Koanga Mogtomo, Alembert Tiabou Tchinda, Tamokou Jean De Dieu, Huguette Magnifouet Nana, Rébecca Madeleine Ebelle Etame, Lucie Biyiti, Rosalie Annie Ngono Ngane

**Affiliations:** Laboratory of Microbiology and Antimicrobial Substances, University of Dschang, P.O. Box 67, Dschang, Cameroon; Laboratory of Microbiology and Food Quality Control, Institute of Fisheries and Aquatic Sciences, University of Douala, P.O. Box 7236, Douala, Cameroon; Laboratory of Biochemistry, University of Douala, P.O. Box 24157, Douala, Cameroon; Laboratory of Phytochemistry, Institute of Medical Research and Medicinal Plants Study, Ministry of Scientific Research and Innovation, P.O. Box 6163, Yaoundé, Cameroon; Laboratory of Phytobiochemistry, University of Yaoundé I, P.O. Box 812, Yaoundé, Cameroon

**Keywords:** *Schefflera barteri*, Acute toxicity, Sub-acute toxicity, Histopathological analysis

## Abstract

**Background:**

The use of herbal medicines as complements or alternatives to orthodox medicines has been on the increase. There has been the erroneous belief that these medicines are free from adverse effects. *Schefflera barteri* is popularly used in the West region of Cameroon for the treatment of various diseases such as diarrhea, spasm, pneumonia and animals bite. Considering the ethnopharmacological relevance of this plant, this study was designed to investigate the possible toxic effects of the stem bark extract of *S. barteri.*

**Methods:**

The extract was prepared by maceration of stem bark dry powder in methylene chloride/methanol mixture. Phytochemical analysis was performed by chemical reaction method. Oral acute toxicity study was carried out by administering single geometric increasing doses (2 to 16 g/kg body weight) of plant extract to *Swiss* albino mice. For sub-acute toxicity study, repeated doses (100, 200, 400 and 800 mg/kg bw) of plant extract were given to *Wistar* albino rats for 28 consecutive days by oral route. At the end of the treatment period, hematological and biochemical parameters were assessed, as well as histopathological studies.

**Results:**

Phytochemical analysis of stem bark extract of *S. barteri* revealed the presence of anthocyanins, anthraquinons and saponins. Acute toxicity results showed that the LD_50_ was greater than 16000 mg/kg. Sub-acute treatment significantly (P < 0.05) increased the level of serum transaminase, proteins and HDL cholesterol. On the other hand, the extract significantly (P < 0.05) reduced the level of leucocytes as well as neutrophils, basophils and monocytes in female. No significant variation of serum creatinine, LDL cholesterol, serum triglycerides as well as liver, spleen, testicles and ovaries proteins was noted. Histopathological analysis of organs showed vascular congestion, inflammation of peri-portal and vacuolization of hepatocytes at the level of the liver. Leucocytes infiltration of peri-portal veins were noticed on lungs and liver cells as well as inflammatory peri-bronchial and basal membranes seminar tube merely joined on lungs and testis respectively.

**Conclusion:**

The results suggest that acute administration of the stem bark extract of *S. barteri* is associated with signs of toxicity, administration over a long duration provokes hepatotoxicity, testes and lungs toxicities.

## Background

In recent times, focus on plant research has increased all over the world and a large body of evidence has collected to show immense potentials of medicinal plants used in various traditional systems [1-3]. The World Health Organization (WHO) estimates that 70 to 80% of the people in developing countries use traditional medicine as a major source of health care. However, many people underestimate the toxicity of natural products and do not realize that these agents could be as toxic or more than synthetic drugs. So far, many plants have been reported to be toxic to both human and animals [1,4]. It should therefore, be emphasized that the traditional use of any plant for medicinal purposes, by no means, warrants the safety of such plant. Plants in folk medicine should therefore, be evaluated for safety or toxicity and necessary recommendations made on their use.

*Schefflera barteri* locally called “Dehethe” in Dschang (Cameroon), “Rukiganame” or “Omwamira” in Uganda, is a shrub belonging to the Araliaceae family. It is distributed throughout Africa’s mountainous forests gallery (Guinea, Sierra Leone, Niger, Uganda…) [5]. In the highlands of the West Region of Cameroon, *S. barteri* is well known for medicinal purpose [6]. The stem bark is widely used for peg fence [7]. From ethnopharmacological data, the leaves or the stem bark are also used to treat diarrhea, spasm, pneumonia and bite from animals. In Uganda, *S. barteri* is reported to reduce dog insensitivity, tiredness and aggressiveness [8]. In spite of the use of *S. barteri* in traditional medicine, scientific data on the plant is limited. Also, systematic evaluation of its toxic effects is lacking. Therefore, this study was designed to investigate the acute and sub-acute toxicity of *S. barteri* stem bark extract.

## Methods

### Plant material

The stem bark of *S. barteri* was collected in Baleveng, Menoua Division, West Region of Cameroon, in March 2010. Identification of the plant was done at the National Herbarium, in Yaounde-Cameroon, using a voucher specimen registered under the reference HNC N° 26155/RSF-Cam.

### Preparation of plant extract

*S. barteri* stem bark were air-dried at room temperature (23 ± 2°C) and milled to coarse particles. A 100 g sample of the powdered material was macerated three times at room temperature in 500 ml of a mixture of methylene chloride/methanol (1:1) for 48 hr, and then filtrated. The filtrate was concentrated using a rotary evaporator (Büchi R200) and the obtained volume was later dried at 50°C to yield 10.05 g of extract. The extract was kept in the freezer at 4°C for further studies. Phytochemical analysis of this extract was performed by standard chemical reaction methods [9].

### Experimental animals

Fifty *Swiss* albino mice (25 males and 25 females, 8 - 10 weeks old) weighing 18-24 g, and 50 *Wistar* albino rats (25 males and 25 females, 8 - 10 weeks old), weighing 120-185 g were used for acute and sub-acute toxicity studies respectively. These animals were bred in the animal house of the University of Dschang and housed in plastic cages under normal laboratory conditions (12 hr light/dark cycle: 23 ± 2°C). They were fed with standard diet. Food and water were given *ad libitum* to all animals used for the experiments. They were handled according to standard protocols for the use of laboratory animals. The studies were conducted according to the ethical guidelines of the Committee for Control and Supervision of Experiments on Animals (Registration no. 173/CPCSEA, dated 28 January, 2000), Government of India, on the use of animals for scientific research.

### Toxicological investigations

#### Acute toxicity study

Fifty mice were randomly allocated into five groups of ten animals each (5 females and 5 males. Group I (Control) was administered orally with vehicle (2.5% (v/v) DMSO/tween 80). Remaining groups (II, III, IV and V)) were administered with geometric increased doses of 2000, 4000, 8000 and 16000 mg/kg body weight of *S. barteri* extract respectively via gastric intubation. Those doses were chosen after several screenings on mice. They were prepared using 2.5% (v/v) DMSO/tween 80 and the administered volume was not more than 1 ml as a unique administration.

The experimental animals were deprived of food for 18 hr prior to extract administration. They were observed continuously for 3 hr thereafter for activity (locomotion), reaction to noise, reaction to pinch, state of excrements and mortality. After this period, the animals were given food and water *ad libitum.* Dead animals in each group were noticed within 48 hr following the administration of the extract. The surviving animals were monitored daily for 14 days for changes in body weight, food and water consumptions [10].

#### Sub-acute toxicity

Fifty albino rats of both sexes were used. They were grouped into five groups of ten animals each (5 males and 5 females). The control group (Group 1) received orally throughout the experiment a solution of 2.5% (v/v) DMSO/tween 80. The test group (2, 3, 4 and 5) received the plant extract at 100, 200, 400 and 800 mg/kg body weight. The administration of various doses of the extract was done by gastric intubation once a day, for 28 consecutive days [11].

#### Food intake and weight gain estimation

Food intake and weight gain was recorded every two days during the experimental time.

#### Sample collection

Rats were fasted overnight on the 28^th^ day and urine was collected from individual metabolic cages, centrifuged and store at +4°C for 24 hours. Upon fasting, the blood samples were collected by cardiac puncture into heparinized and non-heparinized tubes from chloroform anaesthetized rats. Animals were further sacrificed and used for gross pathological examinations and relative organ indices determination.

#### Haematological analysis

The heparinized blood was used for hematological analysis (hematocrit, total red cell (RBCs), total white blood cell (WBCs), lymphocytes, neutrophils, monocytes, eosinophils and basophils) [12].

#### Biochemical analysis

The non-heparinized blood was allowed for complete clotting and then centrifuged at 3000 × g for 5 min. The supernatants (serum samples) were aspired and frozen at -15°C. The serum was assayed for creatinine, aspartate amino transferase (AST), alanine amino transferase (ALT), total cholesterol, high density lipoprotein (HDL), triglycerides and total protein using commercial kits (IMNESCO GmbH, Germany). Urine was assayed for total protein and creatinine using the same commercial kits.

#### Tissues proteins analysis

Immediately after blood collection, the liver, lungs, heart, kidneys, spleen, testis and ovaries were isolated, freed of blood, and weighed using an electronic balance (Mettler PE 160, France). A section of each organ was used for estimation of protein concentration. For this purpose, the homogenate of each organ was prepared in 0.9% NaCl solution at 10% (i.e. 10 g organ in 100 ml of solution). The protein concentrations were determined by the Biuret method [13].

### Histopathological study

Immediately after collecting the blood samples, vascular perfusion was performed for the organ mentioned above and tissue section were further performed (5-micron thickness).

These tissues were further fixed in 10% formalin and then, embedded in paraffin for histopathological analysis. They were routinely stained with haematoxylin and eosin (H & E), and examined under a light microscope (Olympus CH02). Any alterations compared to the normal structures were registered [14].

### Statistical analysis

Results were expressed as mean value ± standard deviation (S.E.M.). Within group, comparisons were performed by the analysis of variance using ANOVA test. Significant difference between control and experimental groups were assessed by Waller Duncan-test.

## Results

### Qualitative phytochemical screening

Phytochemical screening of the stem bark methanol/methylene chloride extract of *S. barteri* revealed the presence of saponins, anthraquinons and anthocyanins while alkaloids, phenols, sterols and triterpenes were not detected.

### Acute oral toxicity

Mice behavior was affected in both sexes by acute treatment with *S. barteri* extract. From 4 000 mg/kg, a reduction of locomotion, reaction to noise and reaction to pinch were noticed. No death were recorded within 48 hours after administration of the extract in animals of both sexes at doses less than or equal to 16 000 mg/kg of body weight.

Food consumption recorded during the periods of observation following the administration of extract is presented in Table 1. A significant (P < 0.05) reduction of food consumption was noticed for all treated mice (i.e. from 2000 mg/kg). The decrease was more pronounced as the doses increased.

**Table 1:** **Effect of daily intake of the methanol/methylene chloride stem bark extract of** ***S. barteri*** **on food consumption in mice according to sex and dose**

**Sexes**	**Dose (g/kg)**	**Food consumption (g)**	
		Week 1	Week 2
Male	0	4.88 ± 0.16^a^	5.38 ± 0.31^a^
	2	4.07 ± 0.13^b^	4.76 ± 0.23^b^
	4	4.08 ± 0.22^b^	4.64 ± 0.12^b^
	8	2.83 ± 0.49^c^	3.58 ± 0.18^c^
	16	2.54 ± 0.38^c^	3.30 ± 0.08^c^
Female	0	5.41 ± 0.26^a^	6.32 ± 0.13^a^
	2	4.50 ± 0.33^b^	5.21 ± 0.44^b^
	4	3.94 ± 0.46^c^	4.70 ± 0.09^c^
	8	3.75 ± 0.47^c^	4.26 ± 0.15^d^
	16	3.71 ± 0.52^c^	4.00 ± 0.88^d^

*Data are expressed as mean ± S.E.M. n = 5. Values for a given group in a line followed by same letter as superscript are not significantly different according to Waller Duncan’s multiple comparison test (P < 0.05).*

**Figure 1:**
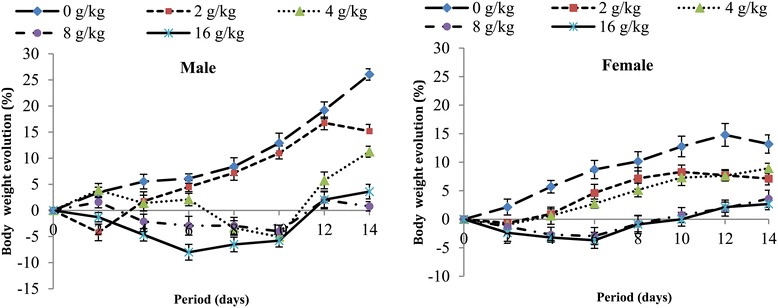
**Body weight evolution of mice in acute toxicity of the methylene/chloride extract of** ***S. barteri.***

The weights of the experimental animals recorded during the two weeks of observation are presented in Figure 1. Mice that received the extract at doses of 8000 and 16000 mg/kg showed a reduction in body weight all over the experimentation time in both sexes, with the reduction being more visible during the first week of experimentation.

### Sub-acute toxicity

#### General signs

No death or significant change in general behavior or other physiological activities were observed during the treatment period either in the controls group or in the extract treated groups.

#### Food intake, weight gain and organ indices

**Figure 2:**
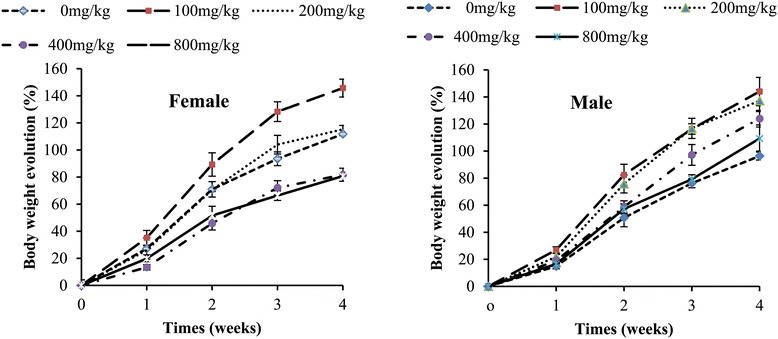
**Body weight evolution of rats in sub-acute toxicity of the methylene/chloride extract of** ***S. barteri.***

The extract did not affect food consumption of male rats but the females were negatively affected during the second week of treatment (Table 2). Throughout the experiment, weight gain decreased in female rats from 400 mg/kg but the males were not affected (Figure 2).

**Table 2:** **Effect of daily intake of the methylene chloride /methanol extract of** ***S. barteri*** **on food consumption in rats according to sex and dose**

**Sexes**	**Doses (mg/kg)**	**Food consumption (g)**			
		**Week 1**	**Week 2**	**Week 3**	**Week 4**
	0	23.28 ± 2.22^a^	28.99 ± 1.82^a^	28.65 ± 3.54^a^	21.97 ± 1.94^a^
	100	22.27 ± 2.21^a^	30.64 ± 0.84^a^	28.39 ± 0.67^a^	24.35 ± 2.57^a^
Male	200	23.16 ± 1.98^a^	31.40 ± 1.33^a^	28.44 ± 2.17^a^	22.05 ± 2.82^a^
	400	21.61 ± 1.99^a^	30.07 ± 1.10^a^	26.93 ± 2.18^a^	22.24 ± 2.50^a^
	800	24.18 ± 1.85^a^	28.09 ± 2.40^a^	26.58 ± 3.18^a^	23.39 ± 2.93^a^
	0	20.85 ± 0.58^a^	26.31 ± 1.41^ab^	23.92 ± 1.18^a^	21.97 ± 2.41^a^
	100	20.24 ± 1.25^a^	27.59 ± 1.82^a^	23.62 ± 1.85^a^	23.35 ± 1.39^a^
Female	200	21.35 ± 1.13^a^	27.64 ± 1.71^a^	23.32 ± 1.61^a^	22.05 ± 2.39^a^
	400	21.89 ± 1.20^a^	24.49 ± 2.35^b^	22.57 ± 3.56^a^	22.24 ± 2.70^a^
	800	21.82 ± 2.07^a^	24.13 ± 1.42^b^	22.15 ± 2.33^a^	23.39 ± 2.75^a^

*Data are expressed as mean ± S.E.M. n = 5. Values for a given group in a line followed by different letter as superscript are significantly different according to Waller Duncan’s multiple comparison test (P < 0.05).*

The results of the effects of *S. barteri* extract on relative organ indices of both male and female rats are summarized in Table 3. There were no significant changes in the lung, kidney, and ovaries or testis to body weight ratios in both groups. However, the extract significantly increased heart and spleen to body weight in both male and female. The significant increase in liver to body weight was observed in male.

**Table 3:** **Relative organ weights indices (g/100g) of rats in sub-acute toxicity of the methylene Chloride/methanol extract of** ***S. barteri***

	**Doses (mg/kg)**	**Liver**	**Spleen**	**Kidneys**	**Lungs**	**Heart**	**Testis/ovaries**
Male	Control	3.68 ± 0.25^b^	0.32 ± 0.06^b^	0.82 ± 0.04^a^	0.67 ± 0.03^a^	0.34 ± 0.03^c^	1.21 ± 0.06^a^
	100	3.84 ± 0.27^b^	0.37 ± 0.03^a^	0.79 ± 0.04^a^	0.68 ± 0.07^a^	0.37 ± 0.04^b^	1.17 ± 0.07^a^
	200	4.10 ± 0.65^a^	0.37 ± 0.03^a^	0.79 ± 0.08^a^	0.68 ± 0.03^a^	0.38 ± 0.02^b^	1.16 ± 0.03^a^
	400	4.11 ± 0.39^a^	0.37 ± 0.05^a^	0.79 ± 0.04^a^	0.69 ± 0.13^a^	0.40 ± 0.05^a^	1.16 ± 0.07^a^
	800	4.63 ± 1.35^a^	0.37 ± 0.06^a^	0.79 ± 0.26^a^	0.70 ± 0.17^a^	0.40 ± 0.06^a^	1.16 ± 0.35^a^
Female	Control	4.19 ± 0.36^a^	0.28 ± 0.08^c^	0.89 ± 0.08^a^	0.69 ± 0.14^a^	0.34 ± 0.03^c^	0.07 ± 0.02^a^
	100	4.20 ± 0.98^a^	0.28 ± 0.06^c^	0.89 ± 0.02^a^	0.68 ± 0.14^a^	0.37 ± 0.04^b^	0.07 ± 0.02^a^
	200	4.32 ± 0.33^a^	0.31 ± 0.04^b^	0.90 ± 0.07^a^	0.68 ± 0.10^a^	0.40 ± 0.02^a^	0.07 ± 0.01^a^
	400	4.30 ± 0.75^a^	0.31 ± 0.02^b^	0.90 ± 0.08^a^	0.69 ± 0.04^a^	0.41 ± 0.03^a^	0.06 ± 0.01^a^
	800	4.31 ± 0.50^a^	0.34 ± 0.07^a^	0.91 ± 0.06^a^	0.69 ± 0.06^a^	0.42 ± 0.05^a^	0.06 ± 0.02^a^

*Data are expressed as mean ± S.E.M. n = 5. Values for a given group in a line followed by different letter as superscript are significantly different according to Waller Duncan’s multiple comparison test (P < 0.05).*

#### Hematological parameters

Hematological analysis indicated that hematocrit, red blood cells count (RBCs), lymphocytes, eosinophils, basophils and monocytes were not affected in males (Table 4). However, total WBCs significantly decreased in both groups with females being more affected. Similarly, neutrophils, basophils and monocytes significantly decreased in female from 200 mg/kg b.w.

**Table 4:** **Hematological parameters of rats in sub-acute toxicity of the methylene chloride/methanol extract of** ***S. barteri***

**Sexes**	**Parameters studied**	**Control**	**100 mg/kg**	**200 mg/kg**	**400 mg/kg**	**800 mg/kg**
	Total RBC (x 10^6^/mm^3^)	3.90 ± 0.22^a^	3.84 ± 0.56^a^	3.80 ± 0.37^a^	3.78 ± 0.55^a^	3.73 ± 0.47^a^
	Total WBC (x 10^3^/mm^3^) 1&110^3^/mm^3^)	226.00 ± 19.50^a^	222.00 ± 16.10^a^	218.00 ± 12.07^a^	214.00 ± 10.25^a^	194.00 ± 16.46^b^
	Hematocrit (%)	45.40 ± 5.63^a^	50.00 ± 2.73^a^	48.40 ± 1.81^a^	49.20 ± 3.96^a^	49.20 ± 3.56^a^
Male	Eosinophils (%)	0.80 ± 0.44^a^	1.00 ± 0.00^a^	1.00 ± 0.00^a^	1.00 ± 0.00^a^	1.00 ± 0.44^a^
	Neutrophils (%)	26.60 ± 4.77^a^	27.00 ± 5.00^a^	27.20 ± 4.14^a^	27.00 ± 7.00^a^	27.60 ± 3.03^a^
	Basophils (%)	0.40 ± 0.54^a^	0.40 ± 0.54^a^	0.41 ± 0.44^a^	0.40 ± 0.44^a^	0.40 ± 0.30^a^
	Monocytes (%)	6.20 ± 1.30^a^	6.20 ± 1.30^a^	5.60 ± 0.55^a^	5.40 ± 0.07^a^	5.40 ± 0.19^a^
	Lymphocytes (%)	66.00 ± 5.00^a^	65.60 ± 2.96^a^	64.80 ± 4.55^a^	61.80 ± 8.13^a^	61.00 ± 2.34^a^
	Total RBC (x 10^6^/mm^3^)	3.39 ± 0.42^a^	3.37 ± 0.24^a^	3.35 ± 0.50^a^	3.37 ± 0.36^a^	3.30 ± 0.40^a^
	Total WBC (x 10^3^/mm^3^)	248.00 ± 13.82^a^	246.00 ± 20.74^a^	244.00 ± 11.40^a^	168.00 ± 8.34^b^	150.00 ± 20.00^b^
	Hematocrit (%)	46.40 ± 2.79^a^	48.60 ± 5.32^a^	48.00 ± 5.43^a^	49.40 ± 3.71^a^	49.80 ± 2.59^a^
Female	Eosinophils (%)	1.40 ± 0.54^a^	1.20 ± 0.44^a^	1.20 ± 0.44^a^	1.200 ± 0.00^a^	1.17 ± 0.00^a^
	Neutrophils (%)	30.00 ± 5.33^a^	29.20 ± 2.50^a^	25.80 ± 1.10^b^	24.60 ± 2.61^b^	23.60 ± 3.03^b^
	Basophils (%)	1.60 ± 0.54^a^	1.20 ± 0.44^bc^	1.00 ± 0.70^bc^	0.80 ± 0.44^c^	0.60 ± 0.54^d^
	Monocytes (%)	6.20 ± 0.77^a^	5.20 ± 0.83^ab^	4.60 ± 1.51^b^	4.20 ± 1.30^b^	4.60 ± 1.51^b^
	Lymphocytes (%)	61.80 ± 5.40^a^	64.00 ± 1.58^a^	65.40 ± 3.58^a^	65.20 ± 2.58^a^	65.20 ± 2.64^a^

*Data are expressed as mean ± S.E.M. n = 5. Values for a given group in a line followed by same letter as superscript are not significantly different according to Waller Duncan’s multiple comparison test (P < 0.05).*WBCs = white blood cells, RBCs = red blood cells.

#### Biochemical parameters

Biochemical values of rats treated with the methylene chloride/methanol extract from *S. barteri* are shown in Table 5. This extract did not affected serum creatinine of animal of both sexes, although, a decrease in the urine creatinine level was noted. Total cholesterol and urinary proteins was not affected in both males and females. Triglycerides decreased significantly in females. HDL-cholesterol increased significantly only in females while LDL-cholesterol decreased significantly in males and females. ALT and AST levels significantly increased in both sexes. Serum proteins increased significantly while hepatic proteins, spleen proteins and testis/ovaries proteins decreased significantly.

**Table 5:** **Biochemical parameters of rats in sub-acute toxicity of the methylene/chloride extract of** ***S. barteri***

	**Parameters studied**	**control**	**100 mg/kg**	**200 mg/kg**	**400 mg/kg**	**800 mg/kg**
	Urinary creatinine (mg/dl)	38.64 ± 3.80^a^	37.76 ± 3.28^a^	37.44 ± 3.14^a^	30.96 ± 1.07^b^	28.96 ± 3.40^b^
	Serum creatinine (mg/dl)	21.04 ± 1.02^a^	24.60 ± 2.47^a^	24.80 ± 4.01^a^	24.80 ± 4.01^a^	24.84 ± 3.20^a^
	Total cholesterol (mg/dl)	122.30 ± 3.37^a^	122.80 ± 3.47^a^	121.92 ± 5.14^a^	121.84 ± 2.65^a^	121.73 ± 1.78^a^
**Male**	HDL cholesterol (mg/dl)	90.42 ± 7.31^a^	90.73 ± 7.18^a^	90.95 ± 6.51^a^	92.86 ± 4.39^a^	93.73 ± 5.12^a^
	LDL cholesterol (mg/dl)	4.15 ± 0.60^a^	4.31 ± 1.56^a^	3.45 ± 1.23^ab^	1.41 ± 0.29^b^	1.40 ± 0.17^b^
	Triglycerides (mg/dl)	141.62 ± 5.38^a^	138.80 ± 8.20^a^	137.60 ± 3.10^a^	137.82 ± 0.35^a^	133.00 ± 3.15^a^
	ALT(U/L)	45.88 ± 1.56^c^	52.00 ± 2.19^b^	54.43 ± 7.36^b^	66.07 ± 5.78^a^	66.24 ± 5.29^a^
	AST (U/L)	7.60 ± 1.10^b^	9.65 ± 0.95^ab^	9.77 ± 0.73^a^	9.79 ± 2.50^a^	9.91 ± 0.63^a^
	Serum protein (mg/dl)	8.35 ± 0.32^c^	8.68 ± 1.22^c^	11.37 ± 1.83^b^	13.73 ± 0.79^a^	14.66 ± 1.76^a^
	Urinary protein (mg/dl)	3.48 ± 0.81^a^	3.42 ± 0.29^a^	3.35 ± 0.45^a^	3.09 ± 0.56^a^	3.06 ± 0.83^a^
	Hepatic protein (mg/g)	562.12 ± 71.22^a^	436.16 ± 39.54^b^	348.67 ± 24.80^c^	273.41 ± 33.30^d^	282.62 ± 32.76^d^
	Spleen protein (mg/g)	332.00 ± 31.87^a^	291.01 ± 27.49^ab^	290.00 ± 32.62^ab^	280.00 ± 26.53^b^	280.00 ± 29.60^b^
	Testis protein (mg/g)	87.10 ± 11.07^a^	84.48 ± 5.43^a^	63.74 ± 13.74^b^	54.69 ± 8.33^b^	53.49 ± 4.25^b^
	Urinary creatinine (mg/dl)	64.32 ± 6.16^a^	63.24 ± 5.06^a^	54.52 ± 2.30^b^	48.68 ± 3.30^c^	48.40 ± 0.35^c^
	Serum creatinine (mg/dl)	31.08 ± 3.00^a^	31.44 ± 1.20^a^	31.44 ± 2.79^a^	31.04 ± 2.10^a^	31.68 ± 1.24^a^
	Total cholesterol (mg/dl)	119.60 ± 2.58^a^	121.73 ± 5.15^a^	121.73 ± 5.15^a^	122.34 ± 3.01^a^	122.59 ± 4.99^a^
**Female**	HDL cholesterol (mg/dl)	71.61 ± 3.35 ^b^	73.26 ± 1.18^b^	77.41 ± 7.43^b^	96.44 ± 2.72^a^	94.44 ± 7.77^a^
	LDL cholesterol (mg/dl)	26.98 ± 5.44^a^	30.98 ± 5.10^a^	28.71 ± 4.39^a^	13.05 ± 2.57^b^	12.16 ± 2.48^b^
	Triglycerides (mg/dl)	96.60 ± 8.90^a^	87.40 ± 7.67^a^	87.00 ± 9.78^a^	71.80 ± 8.08^b^	68.81 ± 4.00^b^
	ALT(U/L)	42.58 ± 9.10^b^	49.57 ± 2.82^a^	49.60 ± 5.37^a^	49.65 ± 8.94^a^	49.91 ± 4.38^a^
	AST (U/L)	9.10 ± 0.80^c^	9.58 ± 0.76^bc^	10.38 ± 1.36^b^	11.96 ± 0.99^a^	12.61 ± 0.99^a^
	Serum protein (mg/dl)	7.13 ± 0.61^b^	8.10 ± 0.20^b^	12.56 ± 0.97^a^	12.79 ± 1.0 ^a^	13.34 ± 0.83^a^
	Urinary protein (mg/dl)	3.30 ± 10.59^a^	3.23 ± 0.37^a^	3.33 ± 0.14^a^	3.35- ± 0.17^a^	3.34 ± 0.56^a^
	Hepatic protein (mg/g)	645.02 ± 19.55^a^	527.62 ± 36.79^b^	380.26 ± 26.11^c^	359.04 ± 43.43^c^	350.88 ± 27.47^c^
	Spleen protein (mg/g)	335.20 ± 34.45^a^	284.00 ± 26.08^b^	288.00 ± 27.27^b^	272.40 ± 37.64^b^	268.00 ± 17.44^b^
	Ovaries protein (mg/g)	546.00 ± 39.77^a^	480.00 ± 84.85^ab^	451.20 ± 31.29 ^b^	449.30 ± 44.18^b^	416.68 ± 24.99^b^

*Data are expressed as mean ± S.E.M. n = 5. Values for a given group in a line followed by different letter as superscript are significantly different according to Waller Duncan’s multiple comparison test (P < 0.05). ALT = alanine transaminase; AST = Aspartate transaminase; HDL = high density lipoprotein; LDL = low density lipoprotein.*

#### Histopathology analysis

**Figure 3:**
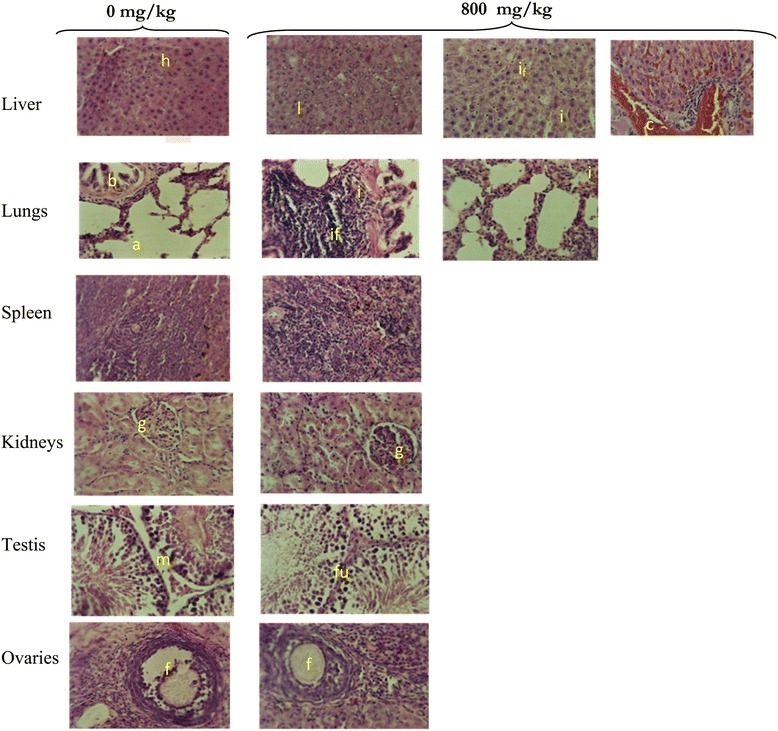
**Histology of organs of the control rats and those exposed of** ***Schefflera barteri*** **for 28 days.** The organs of the rats exposed *S. barteri* extract showed: Light vacuole-like space **(l)** and congestions **(c)** in liver, inflammations **(i)**, peri-portal /peri-bronchial and leucocytes infiltration **(i**_**f**_ **)** in both the liver and lungs, fusion **(f**_**u**_ **)** of basal membranes of seminar tubes **(m)** in testis; congestion of glomeruli and widening of the urinary space, normal spleen and ovary. Histological analysis of the organs of the control rats showed normal structure: hepatocytes **(h)**, alveols **(a)**, bronchi **(b)**, glomeruli **(g)**, membrane, follicle **(f)**.

Histopathological analysis of organs portions after treatment with *S. barteri* stem bark extract revealed varying effect (Figure 3). At the level of the liver, vascular congestion, leucocytes infiltration, periportal inflammation and vacuolization of hepatocytes were noted in both sexes. Leucocytes infiltration and inflammatory peri-bronchial were noticed on the lungs. Inflammatory peri-bronchial and merely joined basal membrane seminar tube were observed on lungs and testis respectively. At the level of the kidney, a congestion of glomeruli and a widening of the urinary space in the 800 mg/kg treated rats as compared to the control group were observed.

## Discussion

Although significant advances have been made in the development and application of *in vitro* toxicity assays, *in vivo* safety evaluation remains the most useful tool for identifying target organ toxicity [15]. The rat has been the species of choice for the vast majority of preclinical toxicology studies performed in the evaluation of pharmaceutical candidates. Recent finding revealed that mouse is a suitable model for very early safety assessment since earlier identification of preclinical toxicities are generally predictive of human toxicity and could save time, money, and effort spent [16].

The acute toxicity study showed no mortality at a dose limit of 16000 mg/kg b.w. by oral administration. The extract *S. barteri* is therefore relatively harmless based on Hodge and Sterner Scale [17]. However, the reduction in mice activity and reaction to noise may be due to depressant and sedative effect on the central nervous system [18]. The reduction of reaction to pinch and reactivity may be due to its inhibitory action on nocireptors or inhibition of the production of algogenic substances (prostaglandins, histamines), or inhibition of the pain signal transmission at the central level [19]. Phytochemical studies of stem bark of *S. barteri* revealed the presence of saponins and such substances may provoke anorexia and weight loss in animals [20]. Their presence in this plant could justify the decrease of both food consumption and weight loss observed during acute toxicity study in mice. Improvement of weight gain noted in the second week may be justified by the biotransformation and elimination of the responsible compounds contained in absorbed extract.

The daily oral administration of the CH_2_Cl_2_/MeOH extract of *S. barteri* stem bark for 28 days did not affect red blood cells, suggesting that oral administration of this extract has no oxygenation and anaemia risk [21,22]. However the decrease in white blood cells indicates that the 28- day’s administration of this extract resulted in the weakening of the immune system [20]. The decrease of neutrophils, basophils and monocytes thus observed may be related to leucocyte infiltrations in the liver and lung revealed by histopathological analysis of these organs.

A significant decrease of hepatic proteins levels was noted, moreover, liver relative weight also increased. These parameters are indicators of hepatic toxicity. Furthermore, a significant increase of AST and ALT in serum was also observed. It is well known that many toxic compounds accumulate in the liver where they are detoxified [23]. Liver damage and its recovery are usually assessed by measuring the level of serum transaminases, particularly ALT. Indeed, changes in their serum level are biological markers of liver dysfunctioning and/or damage [24]. Thus, *S. barteri* extract may be associated with hepatotoxicity. These findings were further confirmed by the histopathological studies on the liver which revealed marked necrosis, vascular congestions, peri- portal inflammations and cell vacuolizations.

Urine creatinine decreased while serum creatinine was not affected. Creatinine is a marker of kidney toxicity, its levels increased in the serum when the cortex and/ or the glomerula are damaged [25]. Glomerula damage is also indicated by the increase of the urine protein levels [26]. No variation of serum creatinine and urine protein levels indicates that the kidneys are normal as shown by histopathological study.

A significant increase in HDL-cholesterol levels in the treated females and reduction in LDL-cholesterol and triglycerides levels in some treated animals were observed. This showed that the extract had some beneficial effects by reducing cardiovascular risk factors, which contribute to death of diabetic patient [27].

Histopathological examination revealed many abnormalities. Vascular congestions on the liver section could be due to the inflammation, blockage or vasoconstriction action of the *S. barteri* extract on the walls of blood vessels. This extract could contain some substances capable of acting like non steroidal anti-inflammatory drugs that provoke hypersensibility reaction which led the lung and liver inflammations observed [28]. The presence of the empty vacuole-like spaces in the hepatocytes could be due to abnormal infiltration of extracellular substances into the hepatocytes or to malfunctioning of the latter [20]. The joined basal membrane of the seminar tube could be due to cellular retraction with reduction of cytoplasmic compounds or cells loss caused by apoptosis [29]. It may also be due to the osmotic gradient modification through the cytoplasmic membrane [30]. The congestion of glomeruli and widening of the urinary space was observed. Drug concentration in the blood is affected by capillary constriction, leading to a decrease in glomerular filtration of that drug which minimizes its effect and protects the tubular cells [31]. This may affect the shrinkage and atrophy of the glomeruli. At the same time, the mesangial cell processes may be retracted due to the contraction of their filaments, which may be stimulated by angiotensin II present in these cells.

## Conclusion

*S. barteri* extract is relatively harmless by acute oral administration. Although sub-acute administration is associated with side effects on the central nervous system, immune system, liver and testis. Therefore, for a *S. barteri* extract based treatment, the dose, frequency and duration of the treatment should be carefully defined to avoid adverse effect of the plant extract.
